# Association Between ADA (Age–D-dimer–Albumin) Score and Chest CT Severity Score in COVID-19 Pneumonia

**DOI:** 10.3390/jpm16020102

**Published:** 2026-02-09

**Authors:** Enrico Maggio, Giacomo Bonito, Alessandra Oliva, Claudio Maria Mastroianni, Riccardo Vezza, Francesco Pugliese, Francesco Violi, Paolo Ricci, Lorenzo Loffredo, Pasquale Pignatelli

**Affiliations:** 1 Department of Medical and Cardiovascular Sciences, Sapienza University of Rome, Viale del Policlinico 155, 00161 Rome, Italy; enrico.maggio@uniroma1.it (E.M.); riccardovezza@libero.it (R.V.); francesco.violi@uniroma1.it (F.V.); 2 Department of Emergency Radiology, Policlinico Umberto I Hospital, Sapienza University of Rome, Viale del Policlinico 155, 00161 Rome, Italy; giacomo.bonito@uniroma1.it (G.B.); paolo.ricci@uniroma1.it (P.R.); 3 Department of Radiological, Oncological and Pathological Sciences, Policlinico Umberto I, Sapienza University of Rome, Viale Regina Elena 324, 00161 Rome, Italy; 4 Department of Public Health and Infectious Diseases, Sapienza University of Rome, 00161 Rome, Italy; alessandra.oliva@uniroma1.it (A.O.); claudio.mastroianni@uniroma1.it (C.M.M.); francesco.pugliese@uniroma1.it (F.P.); 5 Interdisciplinary Department of Well-Being, Health and Environmental Sustainability (BESSA), Sapienza University of Rome, Viale del Policlinico 155, 00161 Rome, Italy; lorenzo.loffredo@uniroma1.it

**Keywords:** ADA score, CT severity score, pneumonia, observational study

## Abstract

**Background**/**Objectives**: This study aims to assess the relation between the ADA score with the severity of pneumonia, as evaluated by chest tomography using a severity score. **Methods**: In this observational study we enrolled 350 consecutive adult patients (≥18 years) with COVID-19-related severe acute pneumonia requiring hospitalization, consecutively admitted to non-intensive care unit (ICU) medical wards from April 2020 to March 2022. A standard high-resolution chest computed tomography (HRCT) was performed in all cases with a multidetector CT scanner without intravenous contrast injection, except in case of suspicion of pulmonary embolism. The ADA score and semi-quantitative 25-point CT Severity Score (CTSS) were calculated for all patients. **Results**: A total of 350 COVID-19 patients (154 males (44%) and 196 females (56%)) were recruited. A logistic regression analysis showed that CTSS is statistically associated with the ADA score (Exp(B): 1.116; 95% CI: 1.027–1.212; *p* = 0.009) and the need for ICU (Exp(B): 8.719; 95% CI: 2.994–25.390; *p* < 0.001), while the linear regression analysis showed a relation between the CTSS and ADA score, GFR and CRP (*p* = 0.003) (predictors: ADA score [β coeff 0.276; 95% CI: 0.041–−0.402; *p* = 0.017], GFR [β coeff −0.219; 95% CI: −0.095–−0.001; *p* = 0.045], CRP [β coeff −0.226; IC 95% −0.077–−0.001; *p* = 0.044]). Furthermore, a ROC curve analysis determined the optimal ADA score cut-off values for predicting severe CT findings at 44.5 (sensibility: 0.971; 1-specificity: 0.670; AUC: 0.750; SE 0.039; *p* < 0.001; 95% CI: 0.674–0.826; Youden’s J index= 0.301). **Conclusions**: This study highlights the potential clinical utility of integrating laboratory- and imaging-based scores for a comprehensive assessment of patients hospitalized with SARS-CoV-2 infection. The combined use of these scores may enable a more accurate identification of patients with extensive pulmonary involvement and an increased prothrombotic burden at hospital admission, facilitating the early recognition of high-risk patients.

## 1. Introduction

The severe acute respiratory syndrome coronavirus 2 (the SARS-CoV-2 virus) is the causative agent of the coronavirus infectious disease-19 (COVID-19 disease), which is associated with a high risk of mortality and morbidity [1]. This disease can cause the well-known pulmonary involvement, but it can also cause thrombotic events, such as stroke, acute coronary syndromes, deep venous thrombosis, and pulmonary embolism in the infected individuals [2]. Indeed, two recent works (a systematic review and a metanalysis including nearly 10 million people) regarding the so-called long COVID showed an association with a higher risk of several cardiovascular diseases, such as thromboembolic disorders, coronary heart disease and stroke compared to non-COVID-19 controls. Interestingly, the pooled risk differences in long COVID cases compared to controls were significantly higher for diseases such as deep vein thrombosis [3]. Increased serum levels of fibrinogen, fibrin degradation products, and D-dimer have been linked with severe disease and increased risk of death [4], and this hypercoagulation state is a key element for the enhanced thrombotic risk occurring in SARS-CoV-2 patients [1]. The severity of COVID-19 is directly proportional to the risk of thrombotic complications [5], and therefore the hypercoagulable state in COVID-19 has serious consequences of morbidity and mortality [6]. The Age–D-dimer–Albumin (ADA) score is a useful tool for identifying high-risk patients for arterial and venous thrombosis in COVID-19 patients and in subjects hospitalized in medical wards. Indeed, this composite score has been shown to be superior to its individual indicators for predicting the development of thrombotic events, even after an external validation [1,7].

Chest computed tomography (CT) is widely considered the best imaging modality for the diagnosis of and follow-up to COVID-19 related pneumonia [8]. Several variants of similar topographical scores were proposed to assess the severity of the lung involvement in COVID-19 infection, such as the total severity score (TSS) and the other two chest CT severity scores (CTSSs) [9,10,11,12]. Previous studies showed a relation between CTSS with the severity of pneumonia and the risk of death in patients with COVID-19 pneumonia [8,9,10,13,14,15,16,17,18,19], thereby validating it as a reliable tool for radiological quantification. Furthermore, the chest CTSS was associated with the prognosis of acute respiratory distress syndrome (ARDS) and COVID-19 pneumonia [8]. Although the pathophysiological connection between COVID-related pneumonia and thrombotic events is well documented, the identification of patients at high risk of developing thrombosis is still inaccurate [20]. Furthermore, it is not clear whether the presence of a thrombotic event could raise suspicion of pneumonia (even in patients with mild respiratory symptoms). This study aims to evaluate the association between the ADA score and the severity of pneumonia, as assessed by chest CT using a validated severity score.

## 2. Materials and Methods

### 2.1. Study Design and Population

This is a single-center, observational, retrospective cohort study performed at Policlinico Umberto I of Rome (the Sapienza university hospital of Rome), in the non-intensive care unit (ICU) medical wards. According to the inclusion/exclusion criteria mentioned below, we enrolled consecutive patients for this study. About the inclusion criteria, in this study, we enrolled only adult patients (≥18 years) with a COVID-19 infection confirmed by our laboratory and with an acute severe COVID-19-related pneumonia that required hospitalization, with or without the need for mechanical ventilation. Severe COVID-related pneumonia has been defined according to the criteria established by the World Health Organization (WHO), with further refinement in the subsequent 2021 guidelines (and reconfirmed by the guidelines up to those of 2025) [21,22,23] (both accessed on 19 January 2026). We enrolled consecutively the patients admitted to medical wards from the second half of April 2020 to March 2022. The Global surveillance for COVID-19 by the WHO Clinical was used to raise and establish clinical suspicion and diagnosis. A patient was defined as a COVID-19-positive case through laboratory confirmation of the SARS-CoV-2 infection.

On the basis of the clinical team’s judgment and of some clinical parameters, we decided whether to perform a CT examination was appropriate or not. All CT scans included in the study were interpreted by a single radiologist to avoid variability between operators.

We decided to include patients who had a chest CT scan at admission (executed in the first 24 h) in our study and to exclude all the ones who had no chest CT at hospital admission in order to avoid/reduce the influence of any therapies implemented to treat pneumonia. After the informed consent for this study was acquired, each participating hospital collected all the useful data (such as demographic information, clinical characteristics, laboratory results, etc.) from COVID-19 patients and sent the data to the Italian National Health Ministry. Laboratory tests routinely performed (including TnThs (high-sensitivity troponin T), NT-pro BNP (N-terminal pro-B-type natriuretic peptide), albumin, and D-dimer) were carried out within 48 h from the admission at the hospital. The primary objective was to evaluate the association between ADA and 25-point CTSS; secondary objectives were determining optimal ADA score cut-off values for predicting high 25-point CTSS, analyzing predictors of high CTSS and the associations with clinical outcomes. We conducted this study after receiving the approval from the Ethics Committee of our center (Azienda Ospedaliera Policlinico Umberto I) (approval date: 07/04/2020; ID Prot. 109/2020) and following the principles of the Declaration of Helsinki.

### 2.2. CT Protocol and Image Analysis

A standard high-resolution chest CT was performed in all cases with a multidetector CT scanner (Somatom Sensation 64; Siemens Healthinners, Forchheim, Germany) without intravenous contrast injection, except in case of suspicion of pulmonary embolism. Images were then reconstructed with a 1 mm slice thickness both with a soft tissue kernel (B30) and a lung kernel (B60) on axial, coronal, and sagittal planes. After each chest CT acquisition, we performed a decontamination with surface disinfection with ethanol (from 62% to 71%) or sodium hypochlorite (0.1%) and a passive air exchange of the CT room.

A semi-quantitative 25-point CTSS, already developed by Pan et al. [24] and validated by Francone et al. [17], was calculated for all patients considering the extent of parenchymal involvement per each of the 5 lobes (0: no involvement; 1: <5% involvement; 2: 5–25% involvement; 3: 26–50% involvement; 4: 51–75% involvement; and 5: >75% involvement). The 25-point CTSS is the result of the sum of each lobar score, giving a range from 0 to 25. The main pattern of lung abnormalities was also described for each patient according to the peer reviewed literature and the glossary for thoracic imaging reported by the Fleischner society on viral pneumonia: ground-glass opacity (GGO), crazy paving, or consolidation. When presented, findings like subpleural lines, traction bronchiectasis, pleural effusion, reversed “halo sign,” and lymphadenopathy were also described. All chest CT examinations were performed using similar acquisition parameters, including slice thickness and reconstruction kernels.

We did not use the data from CT scans performed during the in-hospital patients stay for the analysis.

The electronic medical records of the participants in this study were used to collect data such as demographic characteristics, anamnestic history, baseline clinical information, and laboratory and radiological results. The information about the patients’ comorbidities and the prescribed therapy was also collected. A positive anamnesis for chronic kidney disease (CKD), cardiovascular disease, and cardiovascular risk factors such as hypertension, dyslipidemia, obesity, and diabetes mellitus were defined as previously described [25].

### 2.3. Statistical Analysis

To remain conservative, the sample size calculation was performed assuming a weak Pearson correlation (r = 0.20) between the ADA score and the 25-point CTSS [24]. With a two-tailed α level of 0.05 and 90% power, a minimum of 258 patients was required. To be reasonably certain that we would have enough patients, we chose to enroll more than the calculated number. Normality was evaluated using the Shapiro–Wilk test, while homoscedasticity was assessed by visual inspection of standardized residuals versus standardized predicted values. The residuals showed an approximately constant variance across the range of fitted values with no clear evidence of heteroscedasticity. For the statistical evaluation of the data, we decided to express the categorical variables as count and percentages and the continuous variables as mean and standard deviation (SD). Comparison between groups was performed by the chi-square test and Student’s test, as appropriate, using complete case analysis for the variables involved in each test.

Multivariate logistic regression analysis was performed using complete case (listwise deletion) analysis with a forward selection procedure. Pearson’s correlation analysis or Spearman’s correlation analysis (as appropriate) regarding 25-point CTSS was performed to assess its linear relations with other continuous variables using an available case (pairwise deletion) approach. Multivariable regression analyses were performed using complete case (listwise deletion) analysis. Sensitivity analysis was conducted by repeating the multivariable linear regression analysis using complete case data after the exclusion of influential observations identified by Cook’s distance and leverage values and after assessment of multicollinearity. A *p* value of <0.05 was considered as statistically significant, and all tests we performed were two-tailed. We used SPSS Statistics version 27.0 (IBM Corp., Armonk, NY, USA) to perform the statistical analysis.

The formula that we used for the calculation of normalized ADA score was: ADA score = −0.5 × albumin + 0.4 × D-dimer/100 + 0.3 × age [1].

## 3. Results

**Figure 1:**
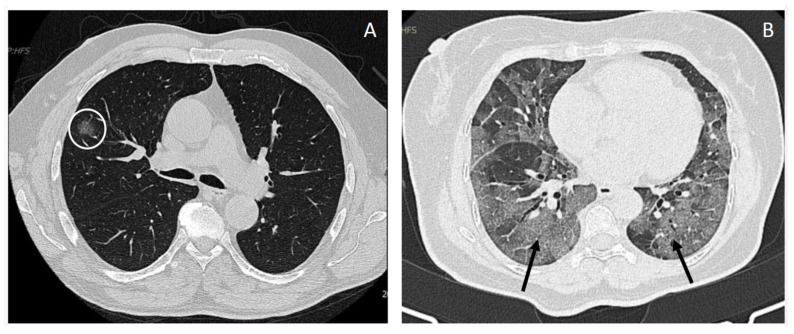
Representative axial chest CT images in lung window showing different degrees of pulmonary involvement. (**A**) Axial CT scan shows a ground glass opacity (white circle) with otherwise preserved lung parenchyma, representative of a mild form of COVID-19 pneumonia (CTSS = 2). (**B**) Axial CT scan shows extensive bilateral crazy paving pattern (black arrows) diffusely involving the lung bases, the middle lobe, and the lingula, suggestive of a severe form of COVID-19 pneumonia (CTSS = 22).

Three hundred and fifty COVID-19 patients (154 males (44%) and 196 females (56%)) were recruited. The median time from admission to CT was 6.8 h. Thrombotic events of the cohort were 48 (13.8%), and the number of patients who died among the general population was 36 (11.3%); CTSS in our cohort had a mean of 8.9 + 6.1 with an elevated prevalence of bronchiectasis (41.7%), parenchymal bands (51.5%), and diffuse multifocal involvement (48.8%). On the other hand, we found a lower prevalence of lymph nodes involvement (12.2%), pleural effusion (8.8%), and pleural traction (4.9%), as well as a rare prevalence of embolism (0.6%) and honeycombing (0.3%). Representative axial chest CT images illustrating mild and severe pulmonary involvement according to the CT severity score are shown in Figure 1.

As shown in Table 1, patients with high CTSS (CTSS ≥ 18) were older, with higher incidence of diabetes mellitus, heart failure, and cancer in the previous five years, as well as the need of intensive care unit, cardiovascular (CV) thrombotic events and death; furthermore, they had higher values of D-dimer and ADA score, as well as lower values of albumin and glomerular filtration rate (GFR). Finally, they had higher incidence of bronchiectasis, pleural traction, parenchymal bands, pleural effusion, diffuse multifocal involvement, and lymph nodes involvement.

A stepwise logistic regression analysis (Table 2) showed a statistically significant association between the CTSS and ADA Score (Exp(B): 1.116; 95% CI: 1.027–1.212; *p* = 0.009) and between the CTSS and the need for admission to intensive care (Exp(B): 8.719; 95% CI: 2.994–25.390; *p* < 0.001).

A linear correlation analysis shows that CTSS correlates with the ADA score, P/F (arterial oxygen partial pressure/fractional inspired oxygen) ratio, SpO_2_ (saturation of peripheral oxygen), respiratory rate, pO_2_, GFR, glycemia, and CRP (C-reactive protein) (Table 3).

The linear regression analysis (Table 4) confirmed the relation between CTSS and ADA score, GFR, and CRP (*p* = 0.003) (predictors: ADA Score [β coeff 0.276; 95% CI: 0.041–−0.402; *p* = 0.017], GFR [β coeff −0.219; 95% CI: −0.095–−0.001; *p* = 0.045], CRP [β coeff −0.226; 95% CI: −0.077–−0.001; *p* = 0.044]). The relationship between the ADA score and the CTSS was confirmed even after adjusting for gender and age (β coeff: 0.722; 95% CI: 0.198–0.960; *p* = 0.003). Sensitivity analysis was conducted by repeating the multivariable linear regression analysis using complete case data after the exclusion of influential observations identified by Cook’s distance and leverage values, and the after assessment of multicollinearity. The results were consistent across all analyses.

**Figure 2:**
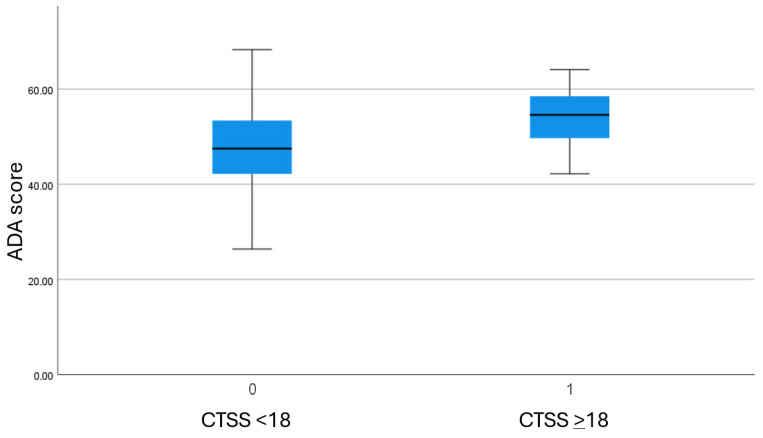
Box-and-whisker plots comparing ADA values between patients with high and low CTSS.

**Figure 3:**
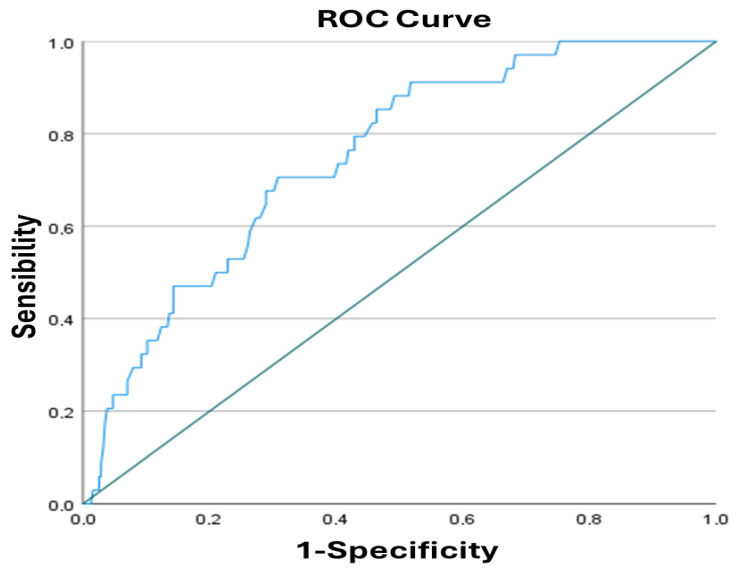
ROC curve analysis to determine optimal ADA score cut-off values for predicting severe CT findings.

Box-and-whisker plots were used to visually compare ADA values between patients with high and low CTSS, allowing clear visualization of medians, interquartile ranges, and dispersion across CTSS categories (Figure 2); the ADA values were higher in patients with CTSS, ≥18, compared with those with CTSS, <18 (median (IQR): 54.6 (58.9–49.6) vs. 47.5 (53.4–42.2)). The ROC curve analysis (Figure 3) determined the optimal ADA score cut-off values for predicting severe CT findings at 44.5 (sensibility: 0.971; 1-specificity: 0.670; AUC: 0.750; SE 0.039; *p* < 0.001; 95% CI: 0.674–0.826; Youden’s J index = 0.301 [moderate performance]) with a typical performance for real-world study according to Youden’s criterion.

## 4. Discussion

This study demonstrates a significant correlation between the ADA score and the CTSS, indicating that higher ADA score values are associated with more severe and extensive radiological involvement.

The ADA score is composed of D-dimer, albumin, and age. These variables, combined in this score, are able to predict venous and arterial thrombotic events, as previously demonstrated. The D-dimer, a component of the ADA score, was previously associated with radiological impairment evaluated using the CTSS in patients with COVID-19 [26].

A retrospective study involving 174 symptomatic SARS-CoV-2 patients demonstrated a correlation between the CTSS and CRP and D-dimer levels, thereby establishing a link between pneumonia severity and the hypercoagulative state induced by the infection. Hence, it is crucial to monitor the prothrombotic state during SARS-CoV-2 pneumonia using tests such as D-dimer and adjust anticoagulant doses accordingly to prevent thrombotic events [13]. Furthermore, even in long COVID, the importance of D-dimer assessment has been emphasized from both respiratory and vascular perspectives. Persistent elevations of D-dimer have been associated with impaired diffusion lung carbon monoxide (DLCO), ongoing symptoms, and an increased thrombotic risk [27,28,29].

Age also plays a significant role in the ADA score, which some authors have associated with the severity of the CTSS. Notably, a retrospective study on 574 COVID-19 patients categorized into six age groups revealed that age can be a significant risk factor for the severity of COVID-19 [18].

Various studies have assessed and validated the role of the CTSS in evaluating the severity of SARS-CoV-2-related pneumonia and short-term prognosis [14], although its role in predicting vascular events remains contentious [30,31]. Clearly, several radiological scoring systems were developed and validated during the pandemic; we chose to focus on the 25-point CTSS because it is a practical and rapid tool that allowed us to evaluate a large number of examinations in a short time, thereby supporting clinicians in assessing disease severity and selecting the most appropriate clinical management strategy [32]. A recent meta-analysis showed that significant pulmonary CT abnormalities remained for up to 2 years post-COVID, especially in patients with severe disease, emphasizing how this pulmonary sequela and related complications could represent an important health problem requiring long-term follow-up, also considering the great number of individuals infected with SARS-CoV-2. [33]. It is also important to consider the different viral variants and their specific characteristics, as confirmed by findings from a recent meta-analysis on pulmonary CT imaging [34].

This study demonstrated a significant association between the ADA score and the CTSS, indicating that more extensive pulmonary involvement is associated with higher ADA score values. This finding is particularly relevant in this patient population, as it underscores the link between pneumonia severity and the risk of venous thromboembolism. Such an association provides a rationale for improved risk stratification and supports a more personalized therapeutic approach, with particular emphasis on the early identification of thrombotic events in order to promptly initiate appropriate anticoagulant therapy [35].

The correlation between the CTSS and respiratory parameters such as P/F ratio, SpO_2_, PaO_2_, and respiratory rate can be explained by considering pneumonia’s role as a pulmonary disease, leading to reduced gas exchange due to alveolar secretions accumulation and a perfusion–ventilation mismatch, particularly in cases of septic shock, resulting in an elevated respiratory rate [36,37,38,39,40]. It is known that there is a correlation between pneumonia severity and elevated CRP levels [41,42,43], and the results are confirmed by showing a correlation between the radiological score explored in this study and CRP levels. Regarding the link between the CTSS and hyperglycemia, it can be attributed to infections and other stressors causing an increase in blood glucose levels as part of the body’s defense mechanism against illness and infection [44,45].

Several studies have evaluated chronic kidney disease’s role as a risk factor for pneumonia, revealing a 1.97-fold higher risk of pneumonia in CKD patients compared to those without CKD [46,47]. This finding could explain the relationship we observed between GFR and the CTSS.

Severe acute respiratory COVID infection is associated with low serum albumin levels, which are linked to a hypercoagulable state [48]. Interestingly, increases in serum albumin levels in patients with COVID-19 are associated with a significant reduction in D-dimer levels, which in turn correlates with clinical improvement and a lower mortality rate [49]. It is likely that this effect may be accompanied by improvements not only in ADA scores but also in radiological findings assessed by CTSS, thus opening new prognostic and therapeutic perspectives. However, this should be explored in future prospective multicenter studies.

The study has several limitations and clinical implications. First, it was conducted at a single center within a specific healthcare setting, which may limit the external validity of the findings and their applicability to different populations or healthcare systems. Second, the number of thrombotic events was relatively low. Third, the decision to perform chest CT at hospital admission was left to clinical judgment, and only patients undergoing CT were included in the analysis. This approach introduces a relevant risk of selection bias, likely enriching the study cohort with patients presenting more severe disease. Fourth, patients who did not undergo CT at admission were excluded, and no direct comparison between included and excluded subjects was performed, further limiting the assessment of potential selection effects. Fifth, the long inclusion period (April 2020 to March 2022) encompassed multiple pandemic waves, different viral variants, and substantial changes in COVID-19 management, including evolving treatment protocols and standards of care. These temporal variations were not explicitly accounted for in the analytical models and may have influenced both clinical outcomes and imaging findings. Finally, the absence of an independent validation cohort prevents confirmation of the robustness and reproducibility of the observed associations.

Taken together, these limitations may restrict the generalizability of our results to the broader population of hospitalized patients with COVID-19 and limit causal inference. Therefore, future multicenter studies with larger sample sizes and external validation cohorts are warranted to confirm and extend our findings.

## 5. Conclusions

This study highlights the potential clinical utility of integrating laboratory- and imaging-based scores for a comprehensive assessment of patients hospitalized with SARS-CoV-2 infection. The significant association observed between the ADA score and the CTSS suggests that their combined use may enable a more accurate identification of patients with extensive pulmonary involvement and an increased prothrombotic burden at hospital admission. Such an integrated approach could facilitate the early recognition of high-risk patients who may benefit from closer monitoring and more intensive diagnostic strategies for thrombotic complications. Further investigations are also needed to assess whether the observed association between the ADA score and CTSS can be extended to other clinical contexts, including non–COVID-related pneumonia.

## Figures and Tables

**Table 1:** Characteristics of patients with low and high CTSS.

N = 350							
	Low CTSS	High CTSS	*p*		Low CTSS	High CTSS	*p*
Male	144/316 (45.6%)	10/34 (29.4%)	0.071	ICU	14/241 (5.8%)	12/25 (48.0%)	<0.001
Age	63 ± 18	73 ± 15	0.002	CV Thrombotic Event	37/315 (11.8%)	11/34 (32.4%)	<0.001
Obesity	9/48 (18.8%)	3/8 (37.5%)	0.231	GFR	69.5 ± 28.3	56.9 ± 24.7	0.039
Dyslipidemia	8/48 (16.7%)	3/7 (42.9%)	0.106	Length of Stay	20 ± 22	27 ± 12	0.132
Hypertension	141/290 (48.6%)	18/33 (54.6%)	0.757	Albumin	38.0 ± 6.2	32.5 ± 4.4	<0.001
Diabetes	36/199 (18.1%)	11/30 (36.7%)	0.019	D-dimer	1183 ± 1241	2639 ± 1518	<0.001
Active Smoking	19/140 (13.6%)	2/22 (9.1%)	0.561	ADA Score	47.7 ± 8.0	54.4 ± 5.8	<0.001
Former Smoking	13/137 (9.5%)	2/18 (11.1%)	0.827	CTSS	7.8 ± 5.4	19.3 ± 1.5	<0.001
Coronary Artery Disease	32/269 (11.9%)	5/26 (19.2%)	0.281	Bronchiectasis	98/258 (38.0%)	23/32 (71.9%)	<0.001
Heart Failure	26/203 (12.8%)	10/30 (33.3%)	0.004	Honeycombing	1/293 (0.3%)	0/33 (0.0%)	0.737
Peripheral Arterial Disease	39/243 (16.1%)	5/25 (20.0%)	0.612	Pleural Traction	12/293 (4.1%)	4/33 (12.1%)	0.043
Atrial Fibrillation	24/290 (8.3%)	5/33 (15.2%)	0.190	Parenchymal bands	145/293 (49.5%)	23/33 (69.7%)	0.028
Transient Ischemic Attack/Stroke	14/270 (5.2%)	1/26 (3.8%)	0.766	Pleural Effusion	20/294 (6.8%)	9/34 (26.5%)	<0.001
Chronic Obstructive Pulmonary Disease	29/290 (10.0%)	6/32 (18.8%)	0.131	Embolism	2/294 (0.7%)	0/34 (0.0%)	0.630
Dementia	22/240 (9.2%)	1/25 (4.0%)	0.383	Diffuse Multifocal Involvement	141/259 (54.4%)	2/34 (5.9%)	<0.001
Cancer in the Last 5 Years	17/243 (7.0%)	5/24 (20.8%)	0.003	Lymphonodes Involvement	30/293 (10.2%)	10/34 (29.4%)	0.001
Death	26/286 (9.1%)	10/33 (30.3%)	<0.001				

**Table 2:** Logistic regression analysis.

N = 266			
	Exp(B)	95% CI	*p*
ADA Score	1.116	1.027–1.212	0.009
ICU	8.719	2.994–25.390	<0.001

**Table 3:** Pearson and Spearman tests.

CTSS			
	N	Correlation	*p*
ADA Score °	349	0.323 **	<0.001
P/F Ratio °	350	−0.479 **	<0.001
SpO_2_ °	280	−0.134 *	0.025
Respiratory Rate °°	127	0.302 **	<0.001
pO2 °	308	−0.225 **	<0.001
GFR °	201	−0.171 *	0.015
Glycemia °°	318	0.245 **	<0.001
TnThs °	191	0.055	0.447
NT-pro BNP °	22	0.228	0.307
CRP °	333	0.128 **	0.020

° Pearson’s correlation was used for these variables. °° Spearman’s correlation was used for these variables. * the correlation has a two-tailed *p*-value of 0.01; ** the correlation has a two-tailed *p*-value of 0.05.

**Table 4:** Multiple linear regression analysis.

N = 251			
ADA Score	β coeff: 0.276	95% CI: 0.041–0.402	*p* = 0.017
GFR	β coeff: −0.219	95% CI: −0.095–−0.001	*p* = 0.045
Respiratory Rate	β coeff: 0.203	95% CI: −0.029–0.735	*p* = 0.069
CRP	β coeff: −0.226	95% CI: −0.077–−0.001	*p* = 0.044

## Data Availability

The original contributions presented in this study are included in the article. Further inquiries can be directed to the corresponding author.
